# Accurate prediction of activity cliff compounds based on bioactivity profiles depends on assay nearest neighbor relationships

**DOI:** 10.1186/s13321-026-01210-9

**Published:** 2026-06-03

**Authors:** Ryuto Abe, Tomoyuki Miyao, Jürgen Bajorath

**Affiliations:** 1 Department of Life Science Informatics and Data Science, B-IT, LIMES Program Unit Chemical Biology and Medicinal Chemistry, University of Bonn, Friedrich-Hirzebruch-Allee 5/6, 53115 Bonn, Germany; 2 Lamarr Institute for Machine Learning and Artificial Intelligence, University of Bonn, Friedrich-Hirzebruch-Allee 5/6, 53115 Bonn, Germany; 3 Graduate School of Science and Technology, Nara Institute of Science and Technology, 8916-5 Takayama-Cho, Ikoma, Nara 630-0192 Japan; 4 Data Science Center, Nara Institute of Science and Technology, 8916-5 Takayama-Cho, Ikoma, , Nara 630-0192 Japan

**Keywords:** Activity cliffs, Machine learning, Molecular representations, Chemical structure, Bioactivity profiles, Data leakage control

## Abstract

The definition of activity cliffs (ACs) depends on compound similarity and activity difference criteria and on activity data types. ACs are usually defined as pairs or groups of structurally similar compounds or structural analogues that are active against the same target, but have large differences in potency (requiring numerical potency values, preferably equilibrium constants). In addition, ACs have also been defined as pairs of structural analogues that are active or inactive in screening assays. In medicinal chemistry, ACs are of particular interest because they often reveal structure–activity relationship (SAR) determinants during compound optimization. In cheminformatics, ACs present challenging test cases for machine learning (ML) and activity predictions because they represent an extreme form of SAR discontinuity in compound data sets. Given their composition, ACs are notoriously difficult to predict based on chemical structure representations. Various attempts have been made to predict compound pairs forming ACs or the activity of AC compounds via ML, often reporting high accuracy. However, in the absence of data leakage from training to test sets, AC prediction accuracy based on chemical structure is low or moderate at best. As an alternative to structural representations, biological/functional compound descriptors might be considered such as biological assay profiles, which have been investigated for other compound activity prediction. In this work, we report the prediction of AC compounds based on bioactivity profiles derived from a compound profiling matrix using data partitioning schemes designed to control information or data leakage during ML. Under these stringent conditions, AC compound predictions based on bioactivity profiles often failed. However, we also observed subsets of highly accurate predictions and explored in detail why these predictions succeeded, but others failed. The analysis revealed a critically important role of assay similarity for successful AC compound predictions. Most profile assays did not measurably influence the predictions. By contrast, accurate predictions mostly depended on the presence of one or at most a few profile assays that were similar to test assays. In most cases, these profile assays could be identified and exploited for predictions by nearest neighbor searching, thus putting ML performance into perspective.

**Scientific contribution**

As an alternative to the generally difficult prediction of ACs based on chemical structure, we introduce prediction of AC compounds based on bioactivity profiles. We show that accurate activity predictions of AC compounds do not depend on the global information content of assay profiles, but are largely determined by nearest neighbor relationships between profile and test assays.

## Introduction

The exploration and exploitation of structure–activity relationships (SARs) in evolving compound series primarily depend on the presence of SAR continuity (when structural modifications lead to moderate changes in biological activity) [1]. By contrast, SAR discontinuity is introduced when small chemical changes lead to unexpectedly large potency alterations [1]. The presence of such “steep SARs” might occasionally lead to “home run” compounds in chemical optimization, but is not always desirable, especially during late stages of optimization efforts when multiple properties must typically be balanced [2]. The extreme form of SAR discontinuity is represented by activity cliffs (ACs), defined as pairs of structurally highly similar compounds (structural analogues) with large potency differences against the same target (or with activity and inactivity) [3–5]. ACs often reveal activity determinants in compounds such as specific R-groups at given substitution sites and are therefore carefully considered in medicinal chemistry when they are identified. Such cliffs can also be formed in other (physico-chemical) molecular property spaces, but ACs represent the most intensely investigated property cliffs. Systematic exploration of ACs based on compound activity data revealed that ~ 20% of compounds across many different activity classes participated in the formation of ACs [4, 5].

While ACs are relevant for medicinal chemistry, SAR discontinuity negatively impacts quantitative SAR (QSAR) modeling, which generally relies on the presence of SAR continuity, and other qualitative or quantitative machine learning (ML) models for activity or potency value prediction [3]. This is the case because these models generally relate structural alterations of compounds to property changes. Accordingly, within this conceptual framework, very similar compounds are expected to have similar activity (which is also intuitive from a chemical or biological viewpoint). Therefore, ACs should generally be difficult to predict.

First attempts to predict individual compounds participating in the formation of ACs or compound pairs forming ACs were made using random forest (RF) [6] and support vector machine (SVM) models [7], respectively. To enable prediction of compound pairs (instead of individual compounds), SVM models employed matched molecular pair (MMP) representations of ACs [8, 9] and newly introduced MMP kernels [7]. An MMP is defined as a pair of compounds with a chemical modification at a single site such as the exchange of substituents [8]. This AC representation termed MMP-cliff [9] was also applied in subsequent AC predictions [10, 11]. As an alternative to SVM models based on MMP kernels, the condensed graph of reaction (CGR) concept was applied to capture structural modifications in ACs for predictions using QSAR or ML models [12]. In ML models, individual compounds or MMPs were typically represented using molecular fingerprint descriptors, preferentially extended connectivity fingerprints (ECFPs) [13]. In addition to classification models for AC prediction, regression models have also been applied to predict the potency of AC compounds [14, 15]. Furthermore, ACs have been predicted using deep learning models such as convolutional and graph convolutional neural networks [16, 17]. Moreover, AC predictions have been extended through generative design of new AC compounds using transformer models [18].

Consistent with the premise that ACs should in general be difficult to predict, as discussed above, different studies have frequently reported the negative impact of ACs in compound data sets on regression and QSAR modeling, molecular property predictions, or property-oriented deep representation learning [15, 19–21]. However, in the AC classification studies discussed above, high median prediction accuracy at the level of 80% or above was typically observed. In a large-scale prediction of ACs across 100 activity classes, using SVM, RF, extreme gradient boosting, fully connected and message passing neural network models, it was shown that memorizing compounds that appeared in training and test data in different ACs (or non-AC compound pairs) was largely responsible for high prediction accuracy [22]. When this form of data leakage was eliminated, global median prediction accuracy was reduced to the 60% level or below, consistent with the anticipated challenges in predicting ACs. This study also showed that AC prediction accuracy did not scale with methodological complexity since SVM models were overall superior to deep neural networks and nearest neighbor classifiers often approached the prediction accuracy of ML models including SVMs [22].

SAR discontinuity can only affect AC predictions if ML models are built based on chemical structure. Accordingly, the comparably low performance of different models using structural descriptors in the absence of compound data leakage [22] indicates that the structural foundation of AC prediction models is their major limiting factor, rather than algorithmic differences. As an alternative, structure-independent models might be considered. Compound activity predictions were also attempted based on biological features. For instance, structural fingerprints were replaced with other fingerprint representations capturing compound assay profiles from high-throughput screening (so-called HTS fingerprints) [23, 24], providing the basis for a structure-independent and functionally-oriented assessment of molecular similarity. In addition, phenotypic assay profiles have been used [25]. Other studies have used compound-induced gene expression signatures or morphological profiles from cell painting assays as biological descriptors for compound activity prediction [26, 27].

While most predictions of ACs have been carried out at the level of compound pairs (that is, ACs versus non-ACs), in this work, we divide  ACs into the individual molecules and carry out predictions of AC compounds. Specifically, we investigate information- and data leakage-controlled AC compound predictions based on bioactivity profiles and analyze factors responsible for the success or failure of different ML models .

## Methods

### Activity cliff definition

#### Structural similarity criterion

Structural similarity was defined based on membership in matching molecular series (MMS), which extend the MMP concept from compound pairs to series of structural analogues [28]. An MMS was defined here as a set of two or more compounds that share a common core structure and differ only by a substituent at substitution site(s). Compounds containing at most 60 non-hydrogen atoms were fragmented according to Hussain and Rea [8] and indexed using RDKit [29]. Eligible chemical transformations were restricted by a fragment-to-size ratio threshold, defined as $({N}_{sub}-{N}_{att})/{N}_{mol}\le 0.2$. Here, ${N}_{mol}$ is the heavy-atom count of the parent molecule, ${N}_{sub}$ is the heavy-atom count of the substituent fragment associated with the transformation, and ${N}_{att}$ is the number of attachment points in the fragment. MMS were then assembled by combining all compounds sharing the same indexed core.

#### Activity criterion

**Fig. 1:**
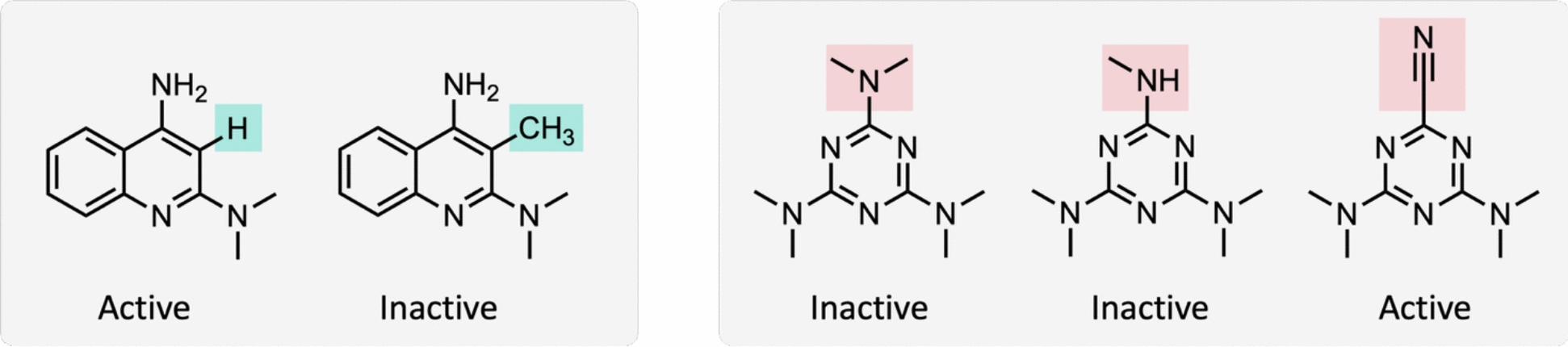
Exemplary activity cliffs. On the left and right, exemplary ACs originating from different MMS are shown. Substituents distinguish different analogues of an MMS. According to the definition applied here, each combination of an active and inactive compound from an MMS represents an AC

In this study, ACs were extracted from assay-based compound profiling matrices (see below), for which numerical potency values were not available. Therefore, different from conventional AC definitions applying potency difference thresholds, ACs were defined here for individual target-based assays based on the binary activity classification active/inactive. Accordingly, an AC was defined as a pair of compounds belonging to the same MMS in which one compound was active and the other inactive [5], as illustrated in Fig. 1. Compared to ACs that are based on potency difference thresholds, this assay readout-dependent definition alters AC characteristics. For instance, binary activity labels are prone to assay noise and assay-dependent binarization criteria. Furthermore, the propensity of ACs is expected to increase because an inactive compound from a given MMS forms ACs with all active compounds from the same series.

### Compound profiling matrix

**Fig. 2:**
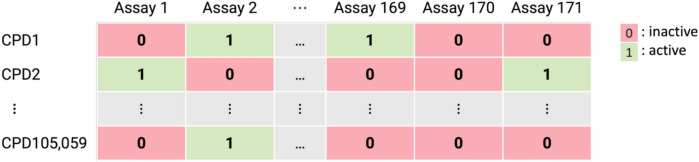
Compound profiling matrix. The composition of a complete compound profiling matrix comprising 105,059 compounds tested in 171 assays is illustrated

Compound profiling matrices encode assay results for compound libraries tested against panels of targets. We used the complete profiling matrix (with no missing values for assay-compound combinations) comprising 171 target-based assays and 224,251 unique compounds reported by Vogt et al. [30]. This profiling matrix was algorithmically extracted [30] from PubChem primary screening assays [31]. Assay results were recorded as binary activity/inactivity readouts. Compounds that were consistently inactive in all assays were removed, resulting in a matrix of 171 assays and 105,059 compounds, illustrated in Fig. 2.

In the profiling matrix, each row represents the “bioactivity profile” of a given compound and each column the “activity vector” of a given assay (this terminology is used in the following). In bioactivity profiles used as a compound representation for ML, each assay represents a unique feature. Compound and assay similarity were quantified by calculating Tanimoto similarity [32] of bioactivity profiles and activity vectors, respectively. The Tanimoto similarity of assays $a$ and $b$ is defined as

1 $$\begin{array}{c}T\left(a,b\right)=\frac{\left|A\cap B\right|}{\left|A\cup B\right|}\end{array}$$

where $A$ and $B$ denote the sets of active training compounds in assays $a$ and $b$, respectively.

### Molecular representations

#### Bioactivity profile

To evaluate model performance, 50 of the 171 target-based assays with at least 500 unique AC compounds were randomly sampled as a hold-out test set. The remaining 121 assays formed the bioactivity profile of the compounds. To investigate the effect of profile composition on compound predictions, we also constructed reduced bioactivity profiles based on assay similarity. For each test assay, pairwise Tanimoto similarity (Eq. 1) was calculated using the activity vector of the test assay and of each of the remaining 121 profiling assays. To avoid data leakage, assay similarities were computed using training compounds only. For a given test assay, a reduced “top_50%” bioactivity profile was generated by selecting the 60 most similar assays and a “top_10” bioactivity profile by selecting only the 10 most similar assays. As a control, we additionally constructed equally sized reduced profiles by randomly sampling of 60 and 10 assays from the set of 121 profiling assays. During model construction, we excluded MMS containing compounds whose annotations were consistently inactive across 121 profiling assays.

#### Structural and combined fingerprints

We used ECFP with bond diameter 4 (radius 2) termed ECFP4 and a constant length of 2048 bits [13] as a structural/topological fingerprint; a standard representation in the cheminformatics field. Combined fingerprints were generated by concatenating a compound’s binary bioactivity profile with its ECFP4 representation.

### Machine learning

#### Classification models

While compound pairs forming ACs have previously been predicted on a large scale [22], in this work, we built binary classification models to predict the activity/inactivity state of individual compounds from ACs. This represents another challenging prediction task because active and inactive compounds are close structural analogues. For each test assay, ML classifiers were trained and evaluated on AC compounds using an intra-series split (see below). Three ML models were constructed including RF [33], extreme gradient boosting (XGB) [34], another decision tree-based approach, and SVM [35]. In each case, three independent models were derived using the bioactivity profile, structural fingerprint, and combined fingerprint representations. All models were refined by fivefold cross-validation on the training data sets. Each model was calibrated on the training data set by optimizing hyperparameter settings via a grid search.

As a control, we also carried out 1-nearest neighbor (1-NN) classification based on assay similarity to examine how well the activity labels of a test assay could be predicted using only a single (most similar) profile assay. For each test assay $t$, we computed assay-assay Tanimoto similarity (Eq. 1) between the activity vector of $t$ and those of 121 candidate profiling assays (denoted $J$), restricting the vectors to the training compound set ${C}_{train}$ obtained for $t$ based on the intra-series split. The 1-NN profile assay ${j}_{NN}$ was defined as the assay with the highest similarity to $t$, i.e., ${j}_{NN}={argmax}_{j\in J} S(t, j)$. Predictions for test compounds $c\in {C}_{test}$ were then generated by directly transferring the binary activity label from assay ${j}_{NN}$, $\widehat{y}(c)={y}_{{j}_{NN}}(c)$.

#### Data partitioning strategy

**Fig. 3:**
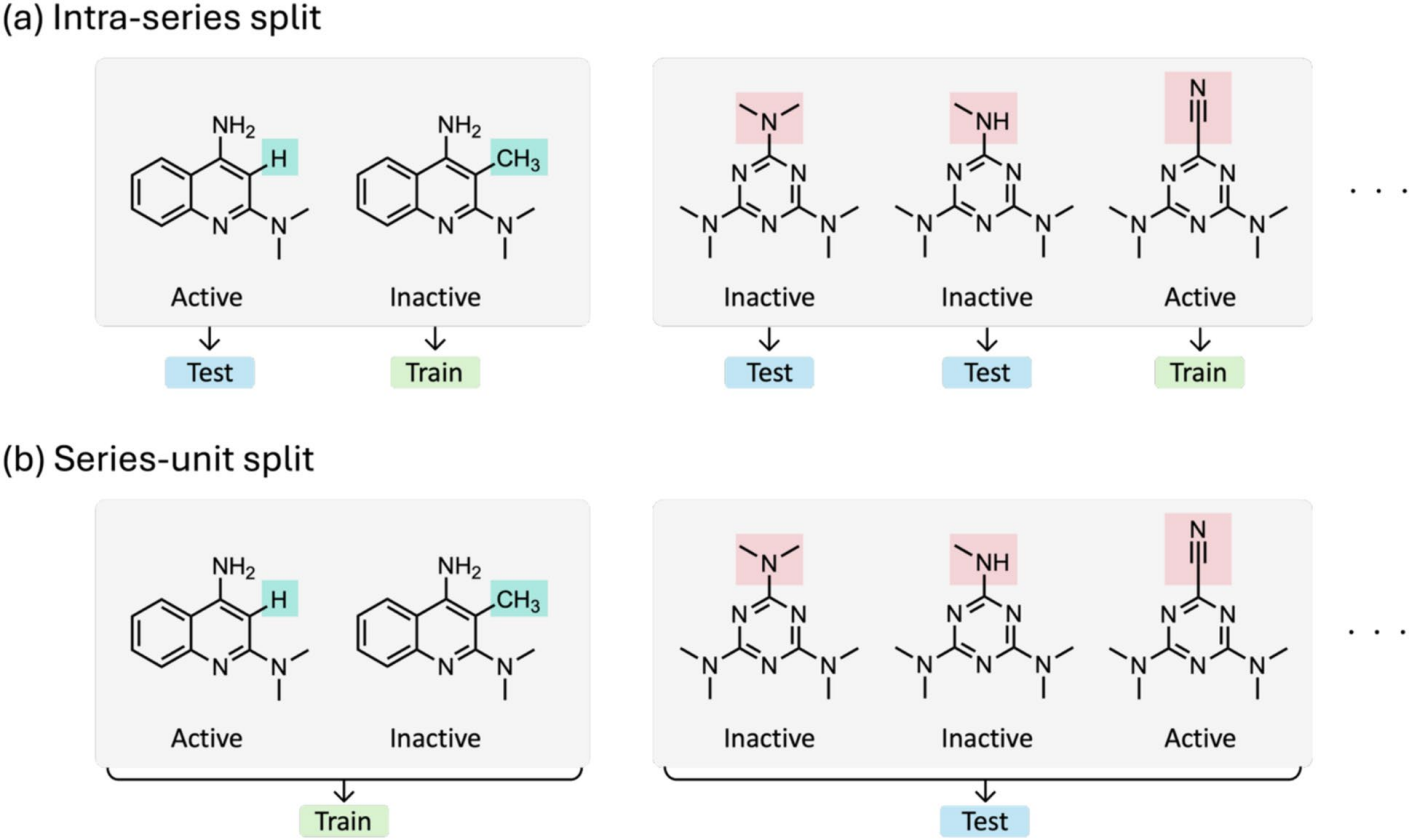
Data partitioning strategies. In **a** and **b**, the intra-series and series-unit splits are illustrated, respectively. Substituents distinguishing different analogues of an MMS and training and test set assignments are color-coded

For each test assay, we assembled a data set of MMS forming ACs. In an “intra-series split”, all active compounds in an MMS were assigned to the training or test data partition and all inactive compounds to the other, as illustrated in Fig. 3a. Thus, for learning, there were no analogues from the same MMS with different class labels (active/inactive) available. However, test compounds included structural analogues from the same MMS with opposite class label, rendering this partitioning scheme a challenging scenario for AC compound predictions. Compared to random data splitting, this partitioning strategy reduced information leakage during training and testing. As an alternative partitioning strategy, a “series-unit split” was evaluated (Fig. 3b). Here, an entire MMS was assigned either to the training or test data partition where analogues had different class labels. This strategy controlled structural data leakage during training and testing. In both cases, the resulting data sets were balanced using different class weights such that minority class samples were assigned higher relative weights during training. While the intra-series split led to variable training and test data ratios, for the series-unit split, a training-to-test data ratio of 80:20% was consistently applied.

#### Performance evaluation

To evaluate model performance, balanced accuracy (BA) [36], area under the receiver-operating characteristic curve (ROC-AUC) [37], and the Matthews correlation coefficient (MCC) [38] were calculated. ROC-AUC was computed as a threshold-independent measure from the predicted scores by evaluating the ROC curve across all possible classification thresholds. The other metrics are defined as follows.

2 $$\begin{array}{c}BA=\frac{1}{2}\left(\frac{TP}{TP+FN}+ \frac{TN}{TN+FP}\right)\end{array}$$

3 $$\begin{array}{c}MCC=\frac{TP\cdot TN-FP\cdot FN}{\sqrt{\left(TP+FP\right)\left(TP+FN\right)\left(TN+FP\right)\left(TN+FN\right)}}\end{array}$$

TP, TN, FP, and FN stand for true positives, true negatives, false positives, and false negatives, respectively.

### Feature importance

To determine the relative importance of assays (features) in a bioactivity profile for predictions, the RF model was analyzed. For each test assay, following model training, the mean decrease in impurity (MDI) was determined as an impurity-based feature importance measure [39, 40]. Accordingly, the importance of a variable ${X}_{m}$ (here, the binary activity readout of profiling assay $m$) was computed by summing the weighted impurity decreases over all nodes $t$ where ${X}_{m}$ is used for splitting, averaged over the ${X}_{T}$ trees:

4 $$\begin{array}{c}Imp\left({X}_{m}\right)=\frac{1}{{N}_{T}}\sum_{T}\sum_{t\in T:v\left({s}_{t}\right)={X}_{m}}p\left(t\right)\Delta i\left({s}_{t},t\right)\end{array}$$

Here, $p\left(t\right)={N}_{t}/N$ is the proportion of training samples reaching node $t$. The decrease in impurity induced by split ${s}_{t}$ at node $t$ is defined as:

5 $$\begin{array}{c}\Delta i\left({s}_{t},t\right)=i\left(t\right)-{p}_{L}i\left({t}_{L}\right)-{p}_{R}i\left({t}_{R}\right),\end{array}$$

with ${p}_{L}={N}_{{t}_{L}}/{N}_{t}$ and ${p}_{R}={N}_{{t}_{R}}/{N}_{t}$, where ${t}_{L}$ and ${t}_{R}$ denote the left and right child nodes, respectively. Importance values were normalized to sum to 1 across all features.

### Statistical analysis

To compare prediction performance for alternative molecular representations, pairwise comparisons were conducted using the two-sided Wilcoxon signed-rank test [41] across the same set of test assays for each model and evaluation metric. To correct for multiple pairwise comparisons, *p* values were adjusted using the Holm-Bonferroni correction. Differences with adjusted *p* < 0.05 were considered statistically significant.

## Results and discussion

### Prediction of activity cliff compounds

**Fig. 4:**
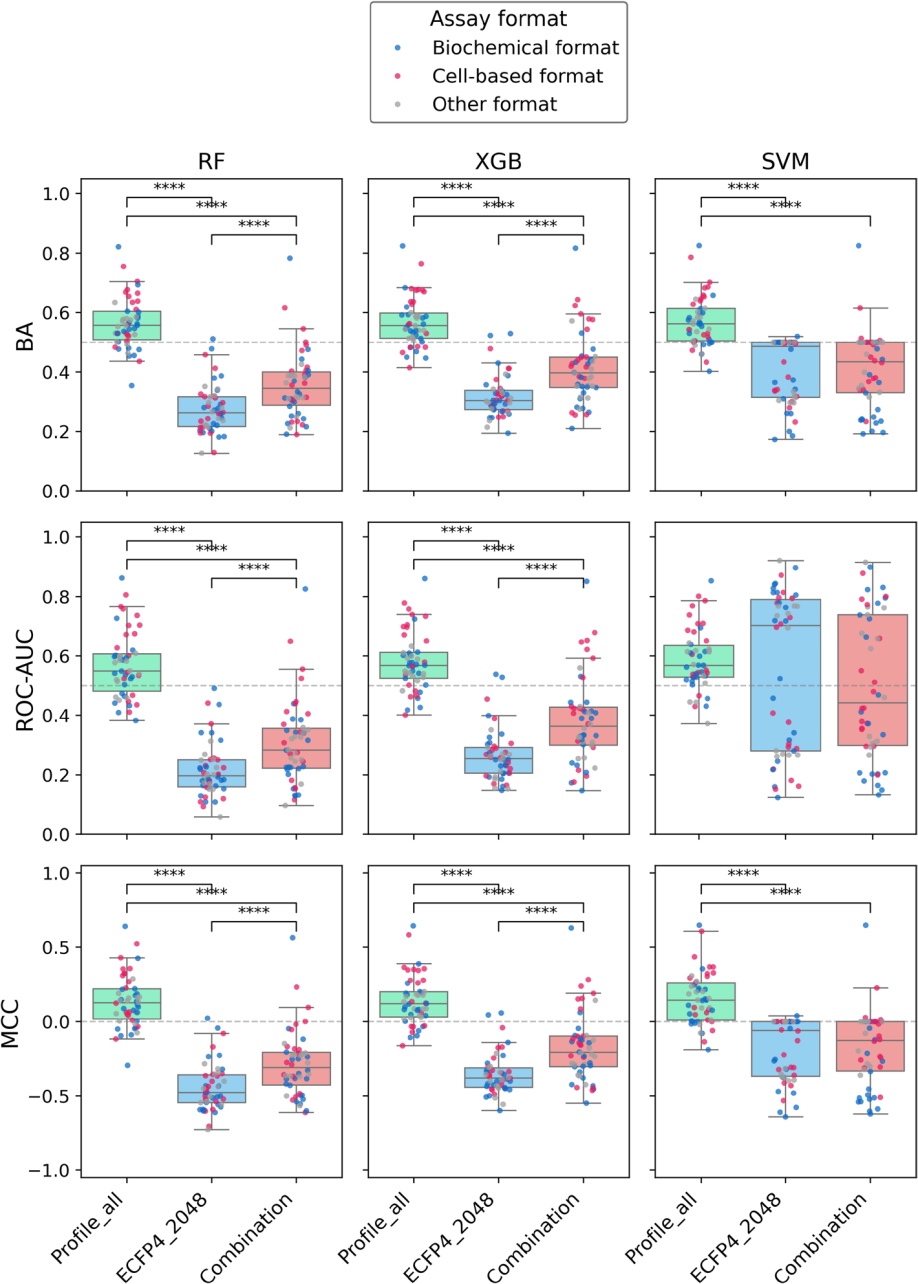
Prediction performance based on intra-series partitioning. Boxplots (box: 1st quartile, median, 3rd quartile; whiskers: +/− 1.5 × interquartile range) report the distribution of BA, ROC-AUC, and MCC values for RF, XGB, and SVM models and 50 test assays for different molecular representations including the bioactivity profile vector for 121 assays (Profile_all), ECFP4 with 2048 bits (ECFP4_2048), and their combination (Combination). In the value distributions corresponding to the boxplots, each dot represents a test assay color-coded by assay format. Statistical significance test: Wilcoxon signed-rank (with Holm-Bonferroni correction). Statistical significance: ****p < 0.0001, ***p < 0.001, **p < 0.01, *p < 0.05, no asterisk p ≥ 0.05 (not significant)

**Fig. 5:**
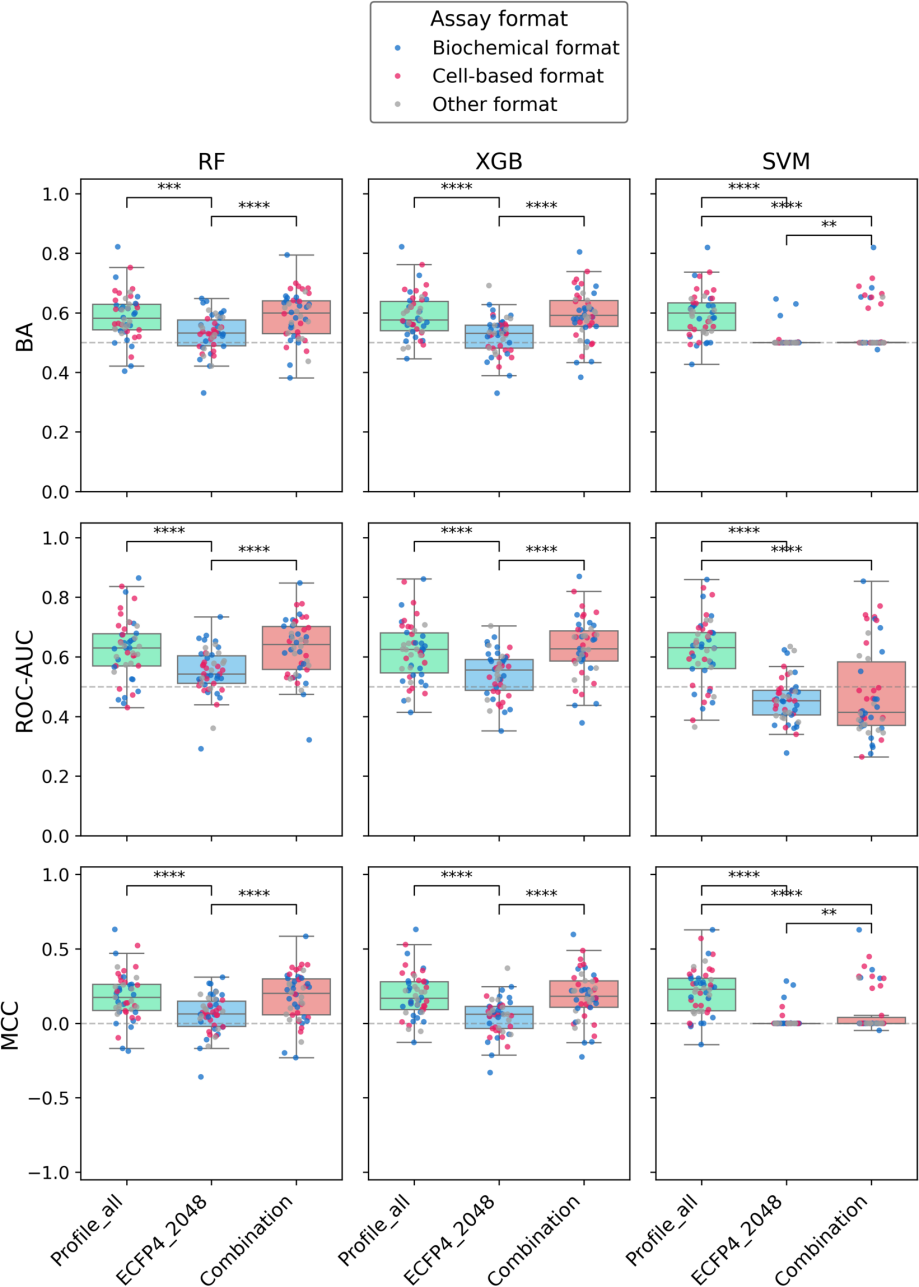
Prediction performance based on series-unit partitioning. Boxplots (box: 1 st quartile, median, 3rd quartile; whiskers: +/− 1.5 × interquartile range) report the distribution of BA, ROC-AUC, and MCC values for RF, XGB, and SVM models and 50 test assays for different molecular representations including the bioactivity profile vector for 121 assays (Profile_all), ECFP4 with 2048 bits (ECFP4_2048), and their combination (Combination). In the value distributions corresponding to the boxplots, each dot represents a test assay color-coded by assay format. Statistical significance test: Wilcoxon signed-rank (with Holm-Bonferroni correction). Statistical significance: ****p < 0.0001, ***p < 0.001, **p < 0.01, *p < 0.05, no asterisk p ≥ 0.05 (not significant)

We carried out systematic predictions of AC compounds applying different methods, data partitioning strategies, and performance measures. Figure 4 and 5 summarize the results of predictions based on intra-series and series-unit partitioning, respectively. For each test assay, compound prediction accuracy was separately determined and the distributions of test assay results were analyzed.

While the predictions of RF and XGB models in Fig. 4 were closely comparable based on the different performance measures, SVM models tended to produce wider value distributions (thus, their predictions were more heterogeneous). For all three methods, only bioactivity profile representations of compounds yielded predictions with better than random accuracy, with median BA and ROC-AUC values of ~ 0.55 and MCC values > 0, whereas the majority of predictions based on the structural ECFP4 compound representation and the combined bioactivity profile/ECFP4 representation failed. The observed differences in prediction accuracy for the alternative molecular representations were consistently statistically significant for RF and XGB models, with ECFP4 yielding the lowest prediction accuracy, and partly significant for SVM models.

Notably, when using the structural representation, either alone or in combination with bioactivity profiles, the accuracy of the majority of predictions was significantly lower than random accuracy (50%); a puzzling observation, at least at a first glance. However, this finding was directly attributable to the intra-series partitioning strategy. In this case, structural analogues from the same MMS in test sets had opposite class labels compared to the corresponding analogues in training sets. Therefore, when ECFP4 features were used, resulting in the detection of high structural similarity between training and test instances, most test compounds were systematically incorrectly predicted, leading to lower than random prediction accuracy.

This explanation was further supported by the results in Fig. 5 based on the alternative series-unit partitioning strategy. Here, the distributions of performance measures were comparably narrow, indicating more homogeneous predictions compared to Fig. 4. Furthermore, the majority of predictions had above-random accuracy (except for SVM models based on ROC-AUC values), with median BA and ROC-AUC values of close to or above 0.6 and MCC values between 0 and 0.5. In this case, the performance of bioactivity profiles and the bioactivity profile/ECFP4 combination was comparable for the decision tree-based models, but not for SVM models (where the combination yielded again lower performance).

The test assay distributions in both Fig. 4 and 5 also revealed that there were no systematic differences in predictive performance for biochemical and cell-based test assays. Notably, the accuracy of AC compound predictions based on the series-unit partitioning strategy that controlled structural data leakage (see Methods) was closely comparable to the accuracy level previously observed when predicting compound pairs forming ACs in the absence of structural data leakage [22], with median BA values of ~ 0.6.

Interestingly, despite the overall low prediction accuracy for the challenging intra-series split, the test assay distributions obtained with all models based on bioactivity profiles contained assays yielding prediction accuracy of ~ 0.7 or above, whereas the structural descriptors consistently failed (Fig. 4). Therefore, we next investigated why bioactivity profiles yielded high prediction accuracy in these cases, but not others.

### Assay similarity

**Fig. 6:**
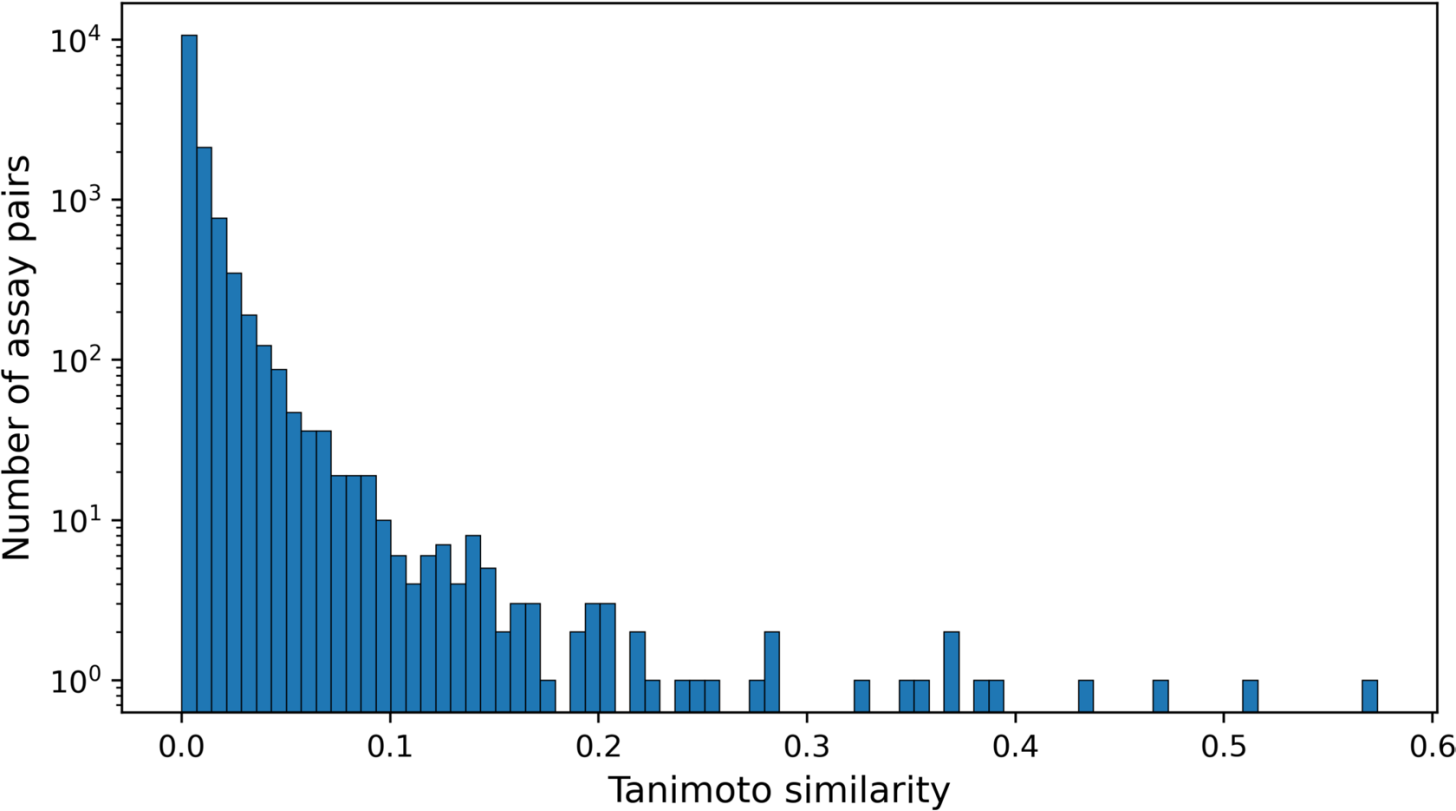
Compound activity-dependent assay similarity. The distribution of pairwise similarity values for all 171 assays contained in the profiling matrix is reported

Initially, we compared the activity vectors of the 171 target-based assays contained in the profiling matrix in a pairwise manner by calculating Tanimoto similarity as a measure of compound activity-dependent assay similarity. Figure 6 shows the resulting distribution of assay similarity values. The majority of assays had low Tanimoto similarity (< 0.1), with a median value close to 0, and only a small subset of the pairs displayed increasing similarity (< 0.6). Thus, compound activity-dependent assay similarity was generally low. This low similarity was consistent with the sparse distribution of active compounds across the profiling matrix. For all assays, the median proportion of active compounds was 0.86%, with a mean of 1.22% and a standard deviation of 2.16%.

### Bioactivity profile information content

**Fig. 7:**
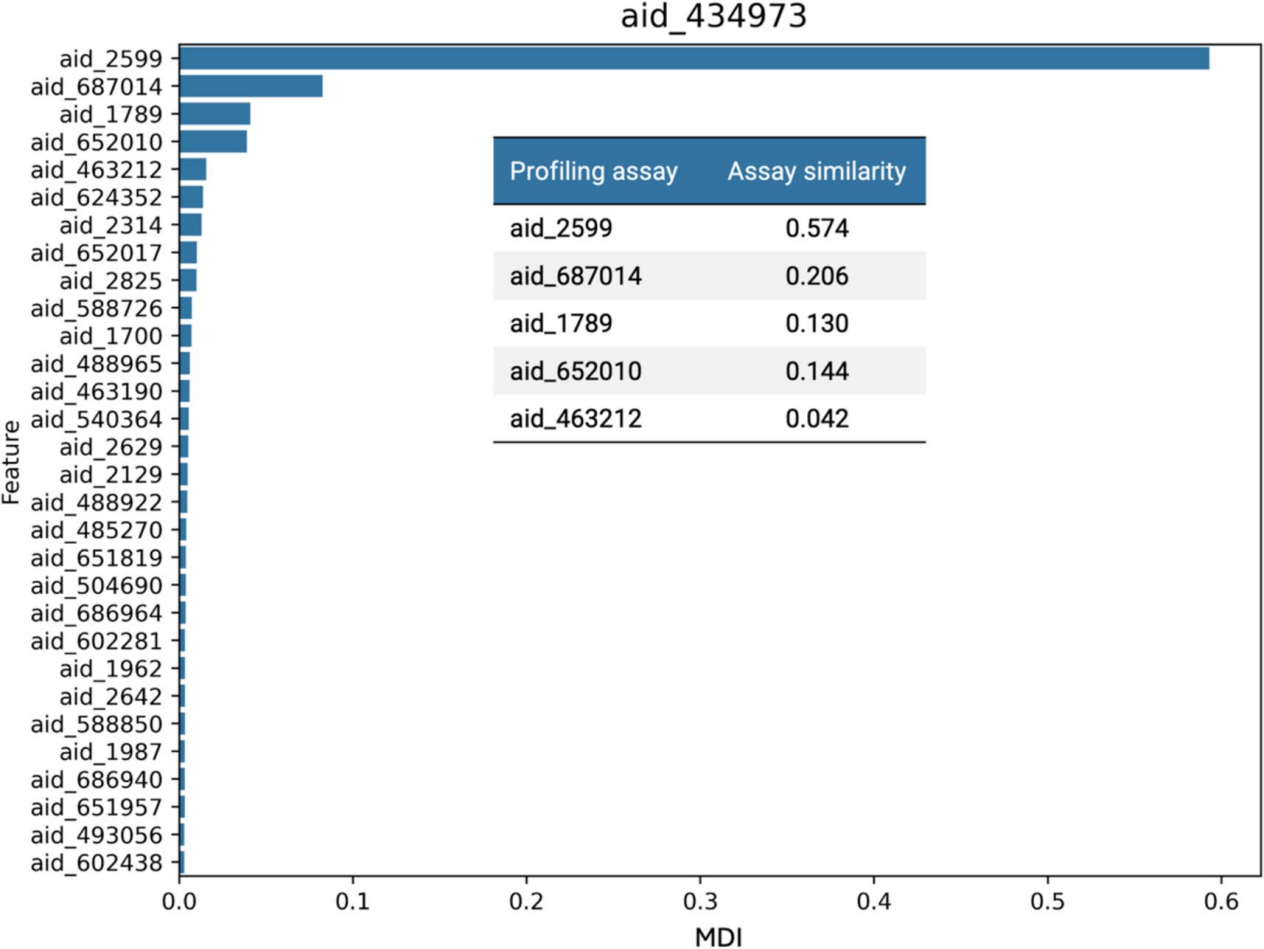
Feature importance and assay similarity. For a representative test assay with high-prediction accuracy of the RF model (BA = 0.82), aid_434973, a biochemical assay for inhibitors of SUMO1/sentrin specific peptidase 7, the top-30 most important profile features are shown. The bar graph reports MDI values for these assays that are identified using PubChem assay identifiers (AIDs). The table insert reports Tanimoto similarity for the top-5 ranked assays compared to the test assay

We next analyzed the information contained in bioactivity profiles of test assays yielding highest prediction accuracy by determining feature (assay) importance for the predictions and similarity between test and profile assays. Interestingly, we found that test assays with high prediction accuracy typically contained only very few assays with significant importance for the predictions, whereas the vast majority of profile assays did not measurably contribute (with feature importance values quickly approaching 0). In fact, in most cases, a single profile assay appeared to dominate the predictions. Figure 7 shows a representative example.

For this test assay with target protein SUMO1/sentrin specific peptidase 7, one bioactivity profile assay (aid_2599) made the by far strongest contribution to the prediction. This assay profile targeted a closely related protein, SUMO-1-specific protease. Calculation of assay similarity revealed that the top-ranked profile assay was most similar to the test assay, as one might expect, given the closely related target proteins. In addition, we observed gradually decreasing similarity for the subsequent assays. Profile assays with feature importance approaching 0 had no detectable similarity to the test assay. Furthermore, in test assays with no predictive performance (that is, random or sub-random prediction accuracy, no similar profile assays were identified. Taken together, the observations made for the test assays with high or no prediction accuracy indicated that one (or very few) most similar profile assays essentially determined the predictions.

### Assay similarity-oriented modeling

We attempted to generalize these findings by globally organizing ML results with respect to assay similarity relationships and by repeating the predictions with specifically modified bioactivity profiles comprising the 60 bioactivity profile assays (50%) or the top-10 profile assays that were most similar to each test assay. As a control, reduced profiles of corresponding size were generated using randomly selected assays.

#### Similarity-based organization of predictions

**Fig. 8.**
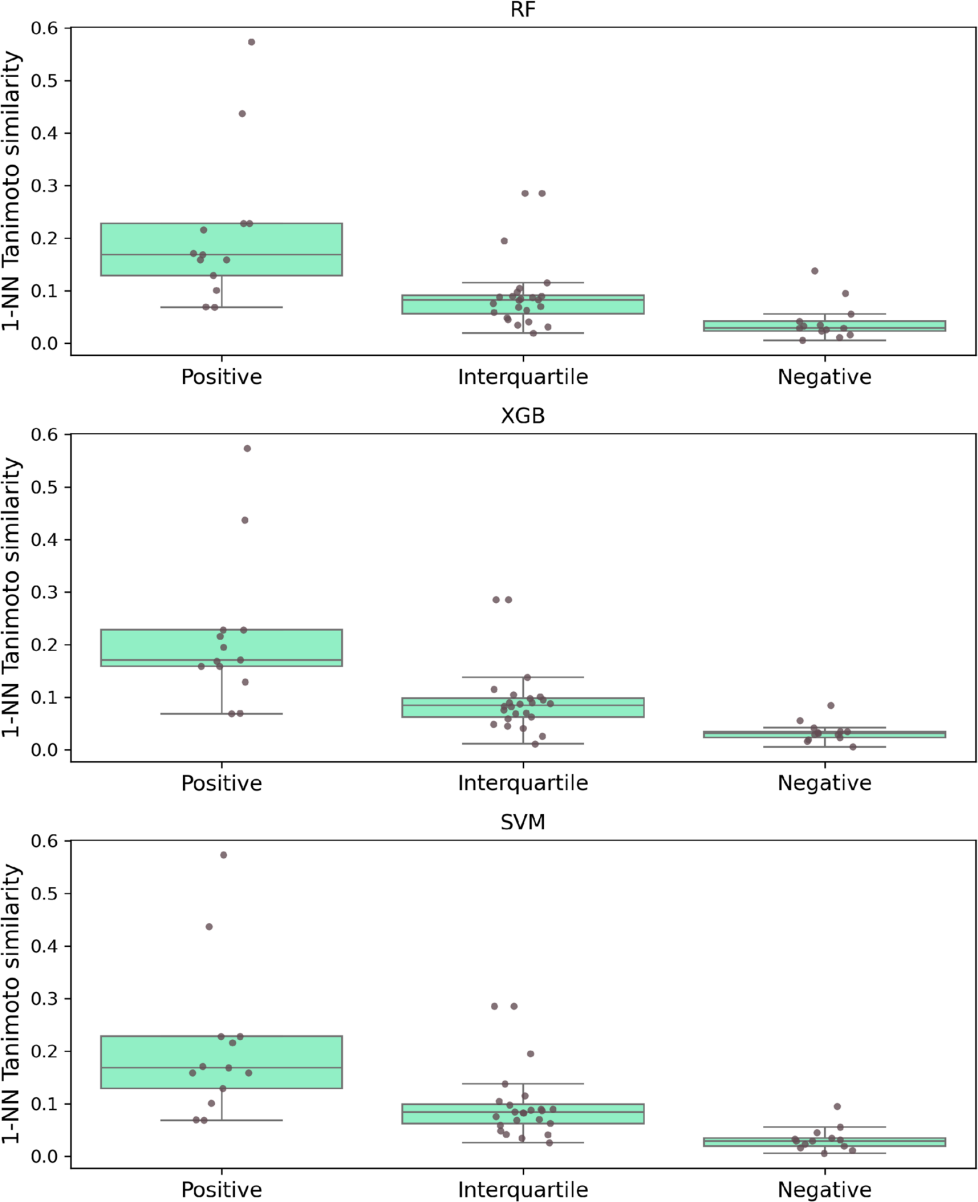
1-Nearest-neighbor assay similarity vs. prediction accuracy. Original predictions of the three models were assigned to three groups of decreasing prediction accuracy based on the statistical distribution of their MCC values, as specified in the text. For each test assay, 1-NN similarity to bioactivity profile assays was determined. Boxplots show the distribution of 1-NN similarity values over the three performance groups

For each model, we organized the predictions of the 50 test assays based on their MCC values in Fig. 4 using the first and third quartiles (Q1 and Q3). Test assays with MCC > Q3 (upper quartile) were labeled “positive” (best predictions), assays with Q1 ≤ MCC ≤ Q3 were labeled “interquartile”, and those with MCC < Q1 (lower quartile) were labeled “negative” (lower than random prediction accuracy). For each test assay, we then computed Tanimoto similarity to all bioactivity profile assays and identified the 1-NN profile assay. Figure 8 shows the distributions of 1-NN similarity values for the three performance groups.

The figure shows a clear trend: as prediction accuracy increases from the negative and interquartile to the positive performance group of test assays, 1-NN similarity to profile assays also increases, hence revealing a general relationship between predictive performance and assay similarity.

#### Similarity-based profile reduction

**Fig. 9:**
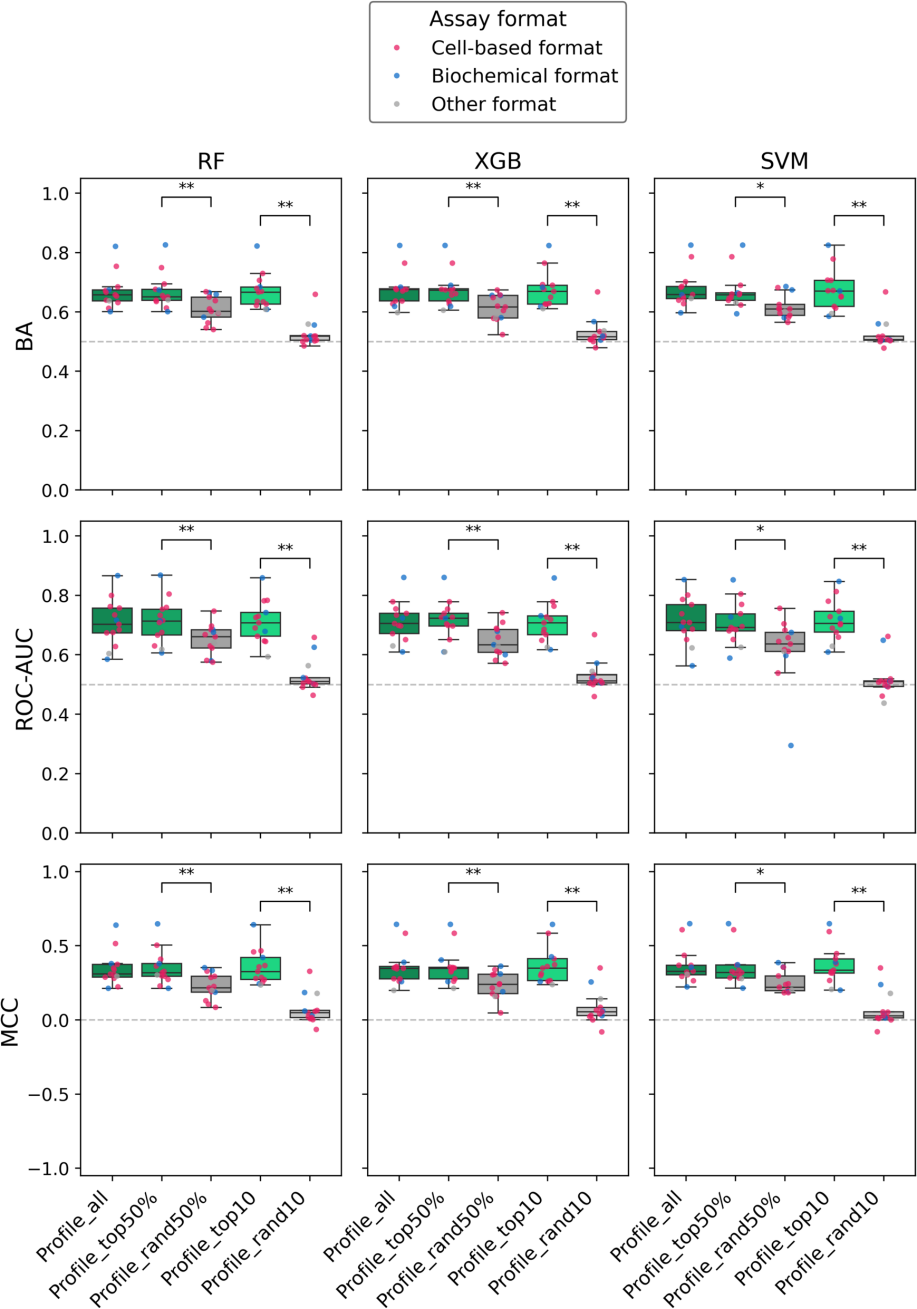
Predictive performance based on reduced bioactivity profiles. For the 13 test assays belonging to the positive performance group, classification results using the three models are compared for the complete bioactivity profile, similarity-based and randomly (rand) reduced profile variants following intra-series partitioning. For clarity, statistical significance (according to Figs. 4 and 5) is reported here only for the corresponding similarity-based and randomly reduced profile variants (Profile_top50% vs. Profile_rand50%, Profile_top10 vs. Profile_rand10)

Considering this relationship, we then generated reduced bioactivity profiles comprising the 60 profile assays (50%) or the top-10 profile assays that were most similar to each of the test assays comprising the positive performance group. As a control, reduced profiles of corresponding size were generated using randomly selected assays. Figure 9 shows the results for the predictions based on reduced bioactivity profiles for the 13 test assays with highest prediction accuracy. For all three models, the accuracy of the original predictions using the complete bioactivity profile (121 assays) was closely comparable to both similarity-based reduced profiles. These findings confirmed that most profile assays did not contribute to the prediction and that a small subset of 10 profile assays most similar to each test assay was sufficient to reach highest prediction accuracy. By contrast, for equally sized randomly reduced profiles, statistically significant and clear reductions in prediction performance were observed. For 10 randomly selected profile assays, the predictive performance was consistently reduced to random prediction accuracy for all models and performance measures. Taken together, these findings confirmed a critically important role of assay similarity for bioactivity profile-based predictions of AC compounds.

#### Nearest neighbor classification

**Fig. 10:**
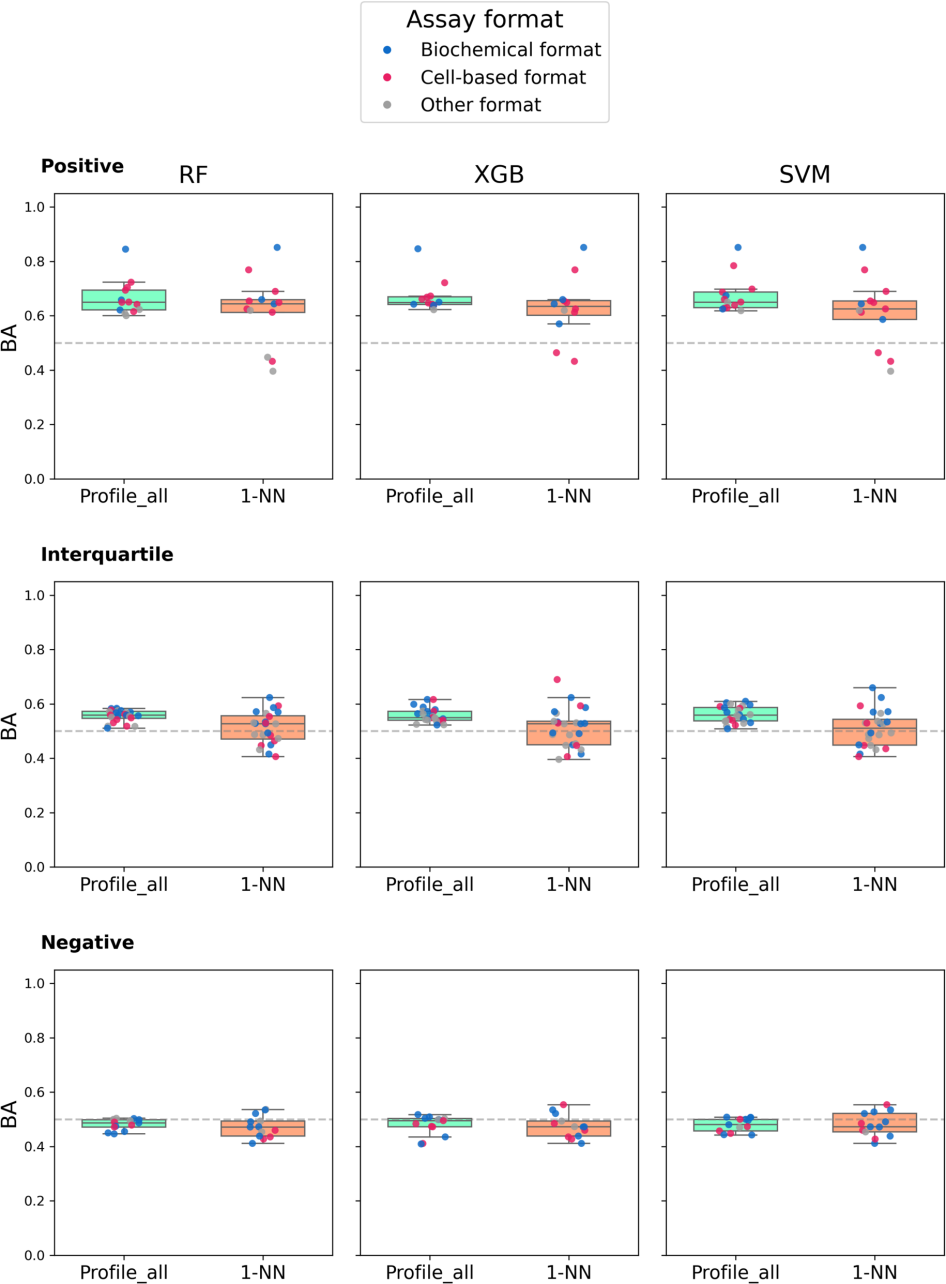
Comparison of the predictive performance on different assay subsets. Prediction accuracy is compared based on complete bioactivity profiles for 1-NN classification and the RF, XGB, and SVM models on three assay subsets including positive, interquartile, and negative assays

Moreover, in light of the observed feature importance dominance of most similar profile assays for accurate predictions, as illustrated in Fig. 7 for the RF model, we reasoned that accurate predictions of AC compounds might largely be determined by nearest neighbor relationships between test and profile assays. Therefore, we also compared the original prediction accuracy for complete bioactivity profiles with 1-NN classification where the activity state of a test compound was assigned to the corresponding state in the most similar profile assay. Figure 10 shows the results for positive, interquartile, and negative assay groups across all three models. For positive assays, 1-NN calculations failed for three test assays (leading to close to random prediction accuracy). However, overall prediction accuracy of 1-NN was only marginally lower than of the three ML models based on complete bioactivity profiles, thus reinforcing the critically important role of assay similarity and NN relationships for successful predictions. By contrast, for interquartile assays, complete bioactivity profiles generally showed slightly higher prediction accuracy than 1-NN classification. Given that 1-NN Tanimoto similarity was lower for interquartile than for positive assays (Fig. 8), this observation indicated that predictions for interquartile assays were not determined by the single nearest neighbor assay alone, but were likely further supported by the combined contributions of multiple profiling assays with moderate similarity. For negative assays, 1-NN Tanimoto similarity was uniformly low (Fig. 8). This was consistent with the close to random performance observed for both 1-NN classification and complete bioactivity profiles, suggesting that neither the nearest neighbor assay nor the combined contributions of multiple low-similarity assays were sufficiently informative for accurate predictions.

## Conclusion

In this work, we have investigated structure-independent predictions of AC compounds using bioactivity profiles as a molecular representation. For our analysis, compound partitioning strategies were devised to control information/data leakage. Under these conditions, prediction performance was overall limited. The intra-series partitioning scheme was particularly challenging, often leading to lower than random prediction accuracy when structural features were used. These findings could be well rationalized considering the presence of close structural analogues with opposing activity class labels in training and test sets. For series-unit partitioning, global prediction accuracy of AC compounds was comparable to the previously observed accuracy of pair-based AC predictions when data leakage was controlled. Overall, predictions of AC compounds based on bioactivity profiles only moderately increased prediction accuracy compared to calculations using structural fingerprints.

However, we also identified a confined subset of test assays with consistently high prediction accuracy and further investigated potential origins of these observations. The Tanimoto similarity of profile assays was generally low, which was at least partly attributable to the prevalence of inactive assay compounds. As a consequence, similarity of test and profile assays was also low for the most part. However, detailed analysis of test assays with high or very low prediction performance revealed that assays yielding meaningful predictions were similar to at least one profile assay, whereas assays with failed predictions lacked similarity to profile assays. These observations indicated an important role of assay similarity for the predictions.

The findings were generalized by repeating predictions following similarity-based or random reduction of bioactivity profiles. In these calculations, the top-10 most similar assays were generally sufficient to meet the prediction accuracy of complete profiles (121 assays), confirming that most (dissimilar) profile assays were irrelevant for the predictions. Moreover, we also showed that the transfer of activity annotations for test compounds from individual 1-NN profile assays was  sufficient to approach or reach the accuracy of most test assays with reasonable or high performance.

Taken together, these findings have important implications for activity predictions based on bioactivity profiles, beyond AC compounds. Provided dense profiling matrices are available as a source of bioactivity profiles, assay-based organization of compound activity readouts makes it possible to determine assay similarity as a prerequisite and indicator of meaningful predictions. Given the relative sparseness of activity annotations in profile assays and their ensuing low similarity, there is no need to assemble large bioactivity matrices for ML. Instead, 1-NN searching may often be sufficient to replace ML models. The simplicity and immediate interpretability of 1-NN searching is an advantage to ML, provided it is sufficiently predictive. However, if profiling assays are sparse, assay NN relationships might be difficult to detect. If no assay similarity is detected, subsequent predictions have a very low probability of success. The importance of assay NN relationships for predictions is also relevant for practical applications such as compound repurposing. If large collections of screening data are available for a compound deck, which is typically the case in the pharmaceutical industry, additional biological activities of compounds that are active in a given assay may be predicted by searching for assays with high (compound activity-based) similarity, without the need for extensive ML campaigns, as suggested by the findings reported herein.

## Data Availability

All data and code are available via the following link: https://uni-bonn.sciebo.de/s/JWfe8ZXnZwLnBSi.

## Abbreviations

AC: Activity cliff
AID: Assay identifier
AUC: Area under the curve
BA: Balanced accuracy
ECFP: Extended connectivity fingerprint
FN: False negative
FP: False positive
MCC: Matthew’s correlation coefficient
MMP: Matched molecular pair
ML: Machine learning
MMS: Matching molecular series
NN: Nearest neighbor
Rand: Random
RF: Random forest
ROC: Receiver operating characteristic
SAR: Structure-activity relationship
SVM: Support vector machine
TN: True negative
TP: True positive
XGB: Extreme gradient boosting
